# Supplementary material for: Integrating genomic structural equation modeling and experimental validation to unravel the genetic basis of male genital lichen sclerosus
Source: Front Immunol. 2026 Jul 16;17:1857945. doi: 10.3389/fimmu.2026.1857945 (PMC13435250; doi:10.3389/fimmu.2026.1857945)
Supplement: Supplementary file 6 [file SupplementaryFile5.zip › result/fuma_PSYCH_factor_sCCA2_focus.chr1.chr1.111741737.113273306.pop1.pdf]

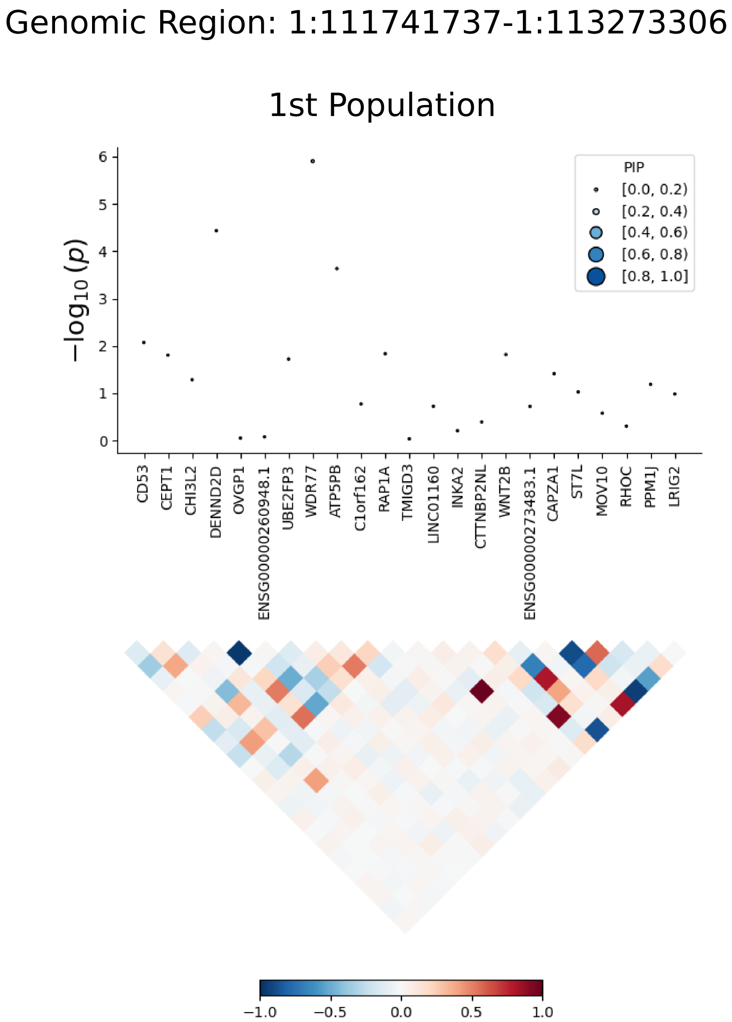
Genomic Region: 1:111741737-1:113273306
1st Population
$-\log_{10}(p)$
0
1
2
3
4
5
6
PIP
[0.0, 0.2)
[0.2, 0.4)
[0.4, 0.6)
[0.6, 0.8)
[0.8, 1.0]
CD53
CEPT1
CHI3L2
DENND2D
OVGP1
ENSG00000260948.1
UBE2FP3
WDR77
ATP5PB
C1orf162
RAP1A
TMIGD3
LINC01160
INKA2
CTTNBP2NL
WNT2B
ENSG00000273483.1
CAPZA1
ST7L
MOV10
RHOC
PPM1J
LRIG2
−1.0
−0.5
0.0
0.5
1.0
